# An aggressive gastric CIC-DUX4 sarcoma surgically resected with multivisceral organs: a case report

**DOI:** 10.1186/s40792-024-02035-0

**Published:** 2024-10-23

**Authors:** Mizuki Koba, Atsushi Takeno, Hiroko Hasegawa, Ryotaro Sakamori, Rei Higashiura, Masaaki Yamamoto, Shinji Tokuyama, Reishi Toshiyama, Kenji Kawai, Yusuke Takahashi, Kenji Sakai, Naoki Hama, Kunihito Gotoh, Takeshi Kato, Yumiko Hirose, Kiyoshi Mori, Masayuki Mano, Motohiro Hirao

**Affiliations:** 1 Department of Surgery, NHO Osaka National Hospital, 2-1-14 Hoenzaka, Chuo-ku, Osaka, 540-0006 Japan; 2 Department of Gastroenterology and Hepatology, NHO Osaka National Hospital, Osaka, Japan; 3 Department of Central Laboratory and Surgical Pathology, NHO Osaka National Hospital, Osaka, Japan

**Keywords:** CIC-DUX4 sarcoma, Stomach, Surgical resection

## Abstract

**Background:**

Capicua transcriptional repressor-double homeobox 4 sarcoma (CDS) is a rare and aggressive malignant soft tissue tumor that typically arises within the soft tissues. We report an exceptionally rare case of a gastric CDS successfully resected despite its extensive invasion into surrounding organs.

**Case presentation:**

A 48-year-old male presented with a progressively enlarging abdominal mass. Upper gastrointestinal endoscopy revealed a large ulcerative tumor on the posterior gastric wall. Biopsy results initially suggested a neuroendocrine cell carcinoma. Contrast-enhanced computed tomography showed a 20 cm tumor protruding from the posterior stomach wall, directly invading the pancreas and colon. We performed a multivisceral resection (stomach, pancreatic tail, spleen, and transverse colon) achieving an R0 resection. Pathological examination of the permanent specimen revealed small round cells with high nuclear-to-cytoplasmic ratios. Immunohistochemical staining confirmed the diagnosis of CDS. The patient recovered well and was discharged on postoperative day 33.

**Conclusions:**

This case report describes the first detailed account of a surgically resected aggressive CDS originating from the stomach.

## Background

Soft tissue sarcomas (STS) are a diverse group of malignant tumors arising from the mesenchyme. The World Health Organization’s 2020 Classification of Tumors recognizes over 80 distinct STS subtypes [1]. This report focuses on a specific subtype: round cell sarcomas within STS. Notably, capicua transcriptional repressor (CIC)-rearranged sarcomas differ clinically, pathologically, and molecularly from Ewing’s sarcoma (EWS). Among CIC-rearranged tumors, the CIC-double homeobox 4 gene (DUX4) gene fusion is the most frequent, resulting from a fusion even between the CIC gene (19q13) and one of two DUX4 retrogenes (4q35 or 10q26) [2].

CIC-DUX4 sarcoma (CDS) is a high-grade, small round cell sarcoma predominantly affecting children and young adults [3]. This rare and aggressive tumor often follows a progressive course, and optimal clinical management strategies for both localized and advanced stages remain unclear. While primarily a soft tissue tumor, CDS can also occur in internal organs, including the brain and bone. The largest molecularly confirmed CDS cohort reported that 86% of cases originated in soft tissues, 3% in bone (all pelvic bones), and 12% in visceral organs, mostly involving the gastrointestinal tract and genitourinary system [2]. Additionally, 5% of cases arose in the retroperitoneum/intra-abdominal/pelvic region, with only one documented case originating from the stomach.

We present an extremely rare case of a surgically resected, aggressive CDS originating from the stomach. To our knowledge, based on a comprehensive PubMed search, this is the first detailed report of such a case.

## Case presentation

A 48-year-old man presented to his primary care physician with tarry stools and abdominal distension. He was subsequently referred to another hospital after an upper gastrointestinal endoscopy revealed a large stomach tumor. Contrast-enhanced computed tomography (CT) showed an 11 cm mass with an uneven contrast pattern within the stomach. Endoscopic ultrasound fine-needle aspiration initially suggested a gastrointestinal stromal tumor (GIST), leading to imatinib therapy. However, subsequent investigations led to a revised diagnosis of neuroendocrine cell carcinoma (NEC). The patient was then transferred to our hospital for specialized treatment.

**Fig. 1:**
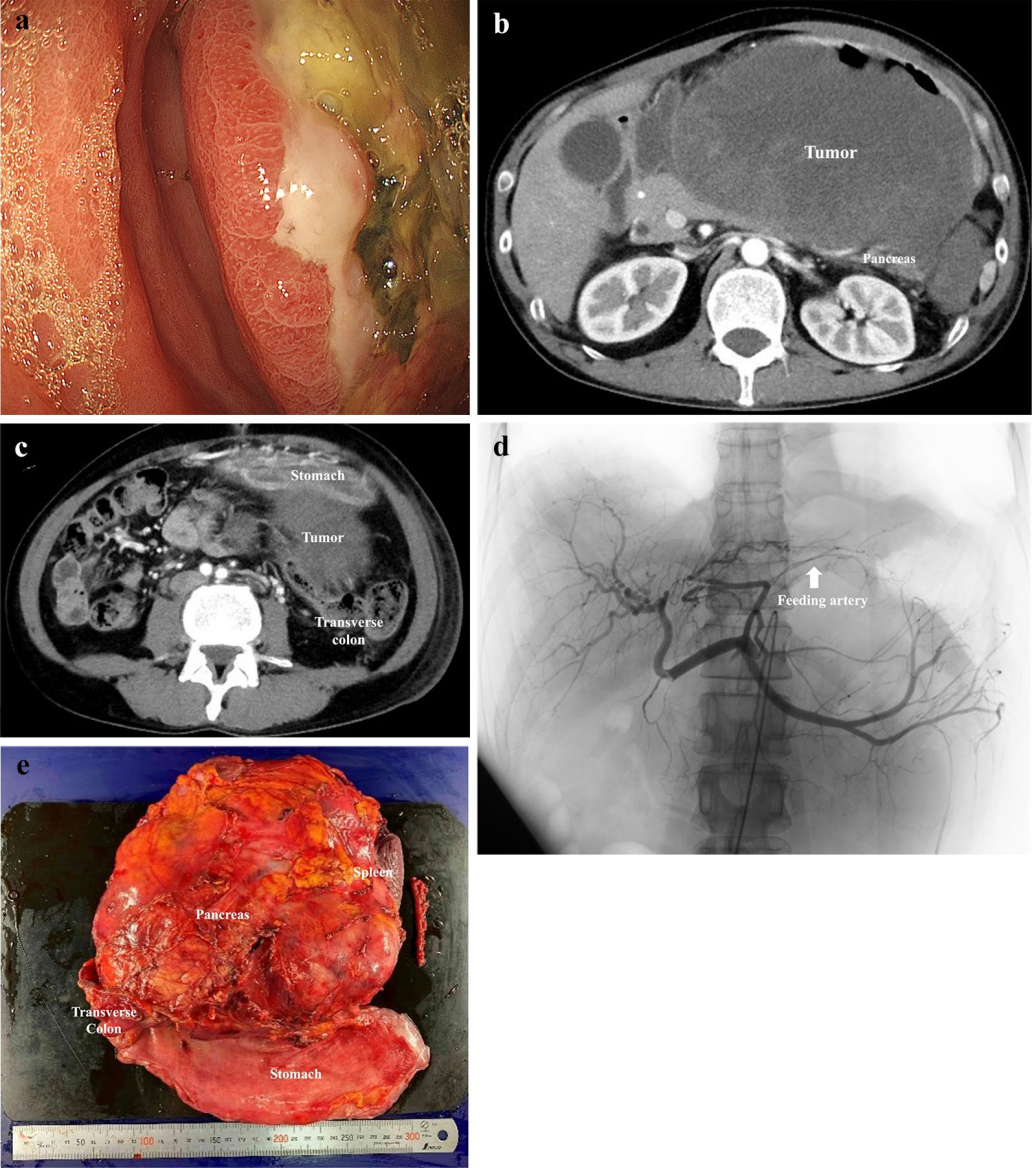
**a** Upper gastrointestinal endoscopy revealed a huge ulcerative tumor with necrosis of the posterior wall of the stomach body. **b** Contrast-enhanced computed tomography revealed a 20 cm in diameter mass protruding dorsally from the posterior gastric wall and invading the pancreas. **c** The tumor invading the mesocolon. **d** Angiography shows the feeding vessel branching from the left gastric artery supplying the tumor. **e** Gross appearance of the resected specimen including stomach, tail of the pancreas, spleen, and transverse colon

Upon transfer, the patient exhibited significant abdominal distention and tenderness. Laboratory findings revealed mild inflammation (white blood cell count: 9000/μL, C-reactive protein: 3.49 mg/dL) and notable anemia (hemoglobin: 7.5 g/dL). Additionally, tumor markers (CA125: 44.4 U/mL and NSE: 82.5 ng/mL) were significantly elevated, along with pancreatic enzyme levels (amylase: 208 IU/L; lipase: 547 U/L). Upper gastrointestinal endoscopy showed a large, ulcerated, and necrotic tumor on the posterior gastric wall (Fig. 1a). Biopsy results weakly indicated CD56 expression, supporting a possible NEC diagnosis alongside the previous GIST suspicion.

Contrast-enhanced CT revealed a substantial 20 cm in diameter mass protruding from the posterior gastric wall invading both the pancreas and the mesocolon. (Fig. 1b, c). This mass had grown by 6 cm in just 2 weeks and appeared to compress the pancreas and potentially directly invade the transverse colon. An enlarged #8 lymph node and a feeding vessel branching from the left gastric artery supplying the tumor were also identified.

The patient required pain management with opioids and repeated blood transfusions due to tumor bleeding. The preoperative diagnosis was cT4b gastric NEC (invading the pancreas and transverse colon), N1 (enlarged #8 lymph node), M0, and cStage IVA. Despite suspected multi-organ invasion, surgical resection was deemed the only option for symptom relief. To minimize intraoperative blood loss, transcatheter vascular embolization of the tumor’s feeding vessel was performed a day before surgery (Fig. 1d).

A midline epigastric incision revealed a large tumor within the distended stomach. Exploration revealed no apparent liver metastases or peritoneal dissemination. The tumor seemed to involve the transverse mesentery and pancreatic tail. We ligated the left gastric and splenic arteries at their origins and used a linear stapler to transect the pancreatic body. A complete en bloc resection of the stomach, pancreatic tail, spleen, and transverse colon was achieved, resulting in an R0 resection (Fig. 1e). The surgery lasted 522 min, with a blood loss of 2520 mL requiring a transfusion of 2600 mL.

**Fig. 2:**
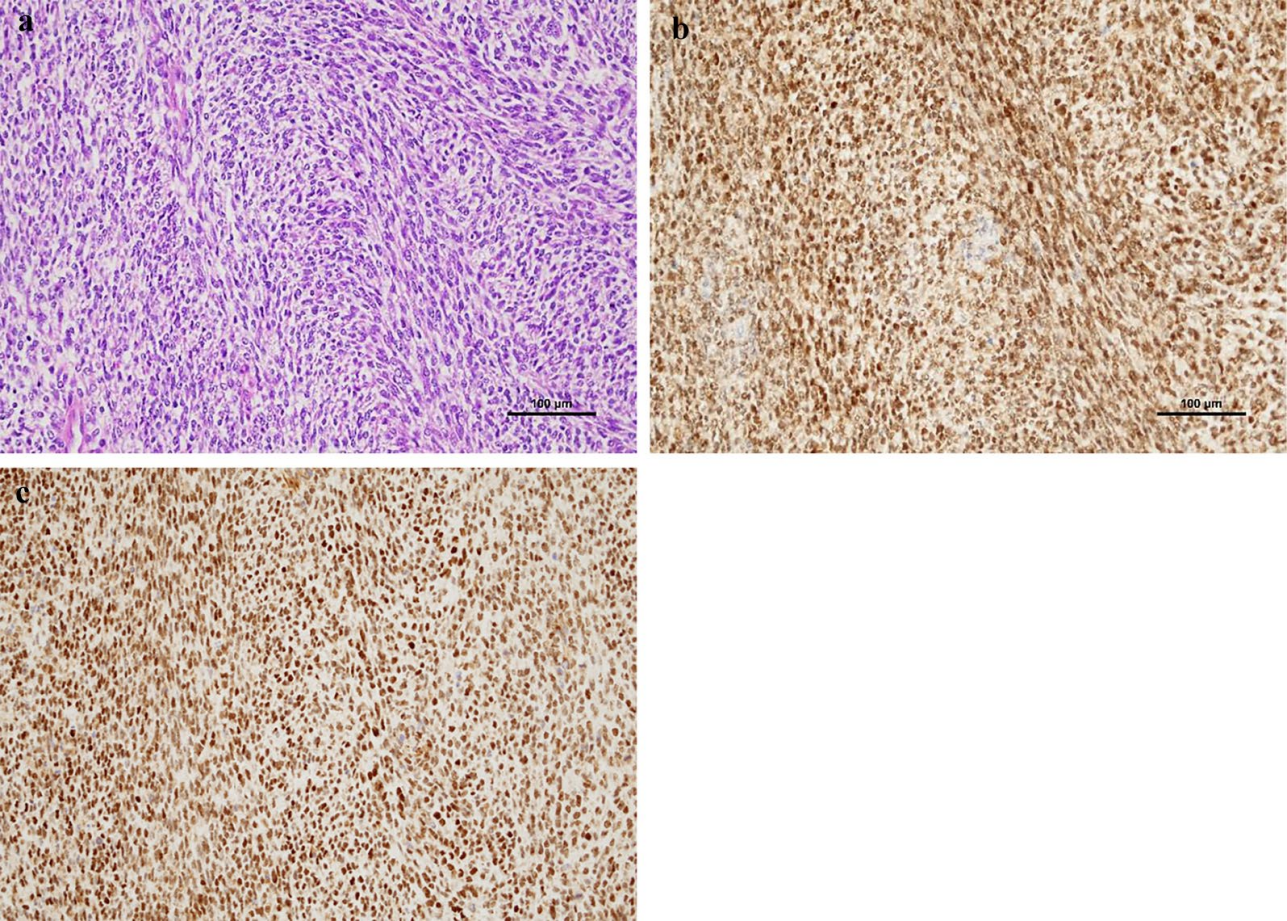
Histopathological and immunohistochemical examination. **a**. Tumor cells show mainly round-to-oval shape or partially spindle shape with a high nucleo-cytoplasm ratio. (× 200 scale). **b**. ETV4 reactivity presents with diffuse staining. (× 200 scale). **c**. DUX4 presents with diffuse nuclear staining. (× 200 scale)

Pathological examination of the permanent specimen revealed sheets of small, round cells with high nuclear-to-cytoplasmic ratios and areas of necrosis. The tumor infiltrated the stomach’s serosal layer, partially invaded the pancreatic parenchyma, and extended into the sub-serosal layer of the colon. Immunohistochemical staining showed partial positivity for CD99 and MDM2, and diffuse positivity for WT1. Markers for GIST (c-kit), epithelial origin (EMA, AE1/AE3), and muscle (Desmin) were negative. Additionally, NUT, P-TRK, SS1, NKX2.2, SATB2, and BCOR were negative, while ERG, ETV4, and DUX4 were positive. Based on these findings, the final diagnosis was CDS (Fig. 2a–c).

The patient developed a Grade IIIa pancreatic fistula [4] and Grade II wound infection postoperatively, both of which were managed conservatively. He recovered well and was discharged on the 33rd postoperative day. He had a very early recurrence of peritoneal dissemination and died 6 months after surgery despite systemic chemotherapy.

## Discussion

CDS primarily occurs in the soft tissues of the trunk and extremities, but it can also affect the gastrointestinal tract. While gastric involvement has been reported, it is exceptionally rare. A study identified only six cases of CDS within the gastrointestinal tract among 111 reported cases, with only one documented case in the stomach; however, detailed information was lacking [2]. To our knowledge, based on a comprehensive PubMed search, this is the first detailed report of a surgically resected CDS originating from the stomach.

The preoperative diagnosis in this case was NEC, highlighting the significant challenges in definitively diagnosing CDS based on small biopsy specimens. Established diagnostic criteria encompass clinical, histological, and immunochemical characteristics.

Yoshida et al. reported that heterogeneous CD99 reactivity, nuclear WT1 expression, and calretinin expression are distinguishing immunohistochemical features of CDS, while NKX2.2 expression is specific to EWS [5]. Specht et al. further suggested that consistent WT1 expression might be a valuable clue for CDS diagnosis [6]. Recently, strong DUX4 immunoexpression was identified as a hallmark of CDS, differentiating it from other round cell sarcomas (EWS, alveolar rhabdomyosarcoma, synovial sarcoma, and desmoplastic small round cell tumor) [7]. The CDS oncoprotein upregulates several ETS family genes (ETV4, ETV1, and ETV5) from the PEA3 subfamily (polyoma enhancer activator [6]. ETV4 expression has also been reported to be a useful tool for diagnosing CDS and distinguishing it from morphological mimics [8]. Our final diagnosis was supported by positivity for both ETV4 and DUX4. While a combination of morphological, immunohistochemical, and molecular findings allows for accurate classification in most cases, a molecular diagnostic approach based on next-generation sequencing technology is becoming increasingly desirable [9, 10].

Surgical resection remains the only definitive treatment for localized, resectable CDS. Chemotherapy, commonly used for EWS, shows limited efficacy in CDS. Connolly et al. advocate for upfront surgery rather than neoadjuvant chemotherapy, citing concerns about the uncertain effectiveness of chemotherapy in localized settings [3]. Given the locally advanced presentation without distant metastases and the uncontrollable tumor hemorrhage in our case, we opted for upfront surgery instead of neoadjuvant chemotherapy. This approach allowed for immediate symptom relief, which was crucial for the patient.

The prognosis for CDS is poor. Yoshida et al. reviewed 20 cases and reported a median overall survival of 12 months, an estimated five-year OS of 17%, and a disease-specific mortality rate of 65% (13 of 20 patients) with 3–19 months of diagnosis [5]. In the largest available series of 115 cases with clinical follow-up data for 57 patients, Antonescu et al. reported two- and five-year overall survival rates of 59% and 49%, respectively [4]. Notably, the overall survival for CDS patients was significantly lower compared to the localized EWS cohort, matched for stage and age, which showed a 76% five-year survival. Approximately 40% of individuals with CDS present with metastasis at diagnosis, primarily involving the lungs. These patients experience significantly worse clinical outcomes compared to those with localized disease. Due to the rarity of CDS, there is no established consensus on treatment protocols. Prospective, multi-institutional clinical trials are urgently needed, along with the creation of a prospective registry. Such resources would be instrumental in better characterizing these tumors and refining optimal treatment strategies [11].

## Conclusions

This report describes an exceptionally rare case of a patient with a gastric CDS that exhibited aggressive growth and invaded the surrounding organs. Despite extensive local involvement, the tumor was successfully resected en bloc with multiple visceral organs.

## Data Availability

Data sharing does not apply to this article as no datasets were generated or analyzed in the current study.

## Abbreviations

CDS: CIC-DUX4 sarcoma
CIC: Capicua transcriptional repressor
CT: Computed tomography
DUX4: Double homeobox 4 gene
GIST: Gastrointestinal stromal tumor
LUAE: Left upper abdominal resection
NEC: Neuroendocrine cell carcinoma
EWS: Ewing’s sarcoma
STS: Soft tissue sarcomas
